# Cell energy metabolism and bone formation

**DOI:** 10.1016/j.bonr.2022.101594

**Published:** 2022-05-27

**Authors:** Rubens Sautchuk, Roman A. Eliseev

**Affiliations:** Center for Musculoskeletal Research, University of Rochester School of Medicine & Dentistry, 601 Elmwood Ave, Rochester, NY 14642, United States

**Keywords:** Osteogenic lineage, Bone, Bioenergetics, Mitochondria, Glycolysis

## Abstract

Energy metabolism plays an important role in cell and tissue ability to effectively function, maintain homeostasis, and perform repair. Yet, the role of energy metabolism in skeletal tissues in general and in bone, in particular, remains understudied. We, here, review the aspects of cell energy metabolism relevant to bone tissue, such as: i) availability of substrates and oxygen; ii) metabolism regulatory mechanisms most active in bone tissue, e.g. HIF and BMP; iii) crosstalk of cell bioenergetics with other cell functions, e.g. proliferation and differentiation; iv) role of glycolysis and mitochondrial oxidative phosphorylation in osteogenic lineage; and v) most significant changes in bone energy metabolism observed in aging and other pathologies. In addition, we review available methods to study energy metabolism on a subcellular, cellular, tissue, and live animal levels.

## 1 Introduction

The bone field is lagging behind neuroscience, cardiovascular, and other fields in understanding the role of cell energy metabolism in tissue physiology and pathology. Integrating knowledge of energy metabolism/bioenergetics in osteogenic lineage cells into various aspects of bone research will be very beneficial and far-reaching as has been in other fields mentioned above. Therefore, the goal of this review is to summarize what is known about cell energy metabolism regulation, crosstalk with other cell functions, changes observed in pathologies, and study methodologies as relevant to osteolineage. We will focus on bone forming osteoblasts (OB), their precursors, bone marrow stromal (mesenchymal stem) cells (BMSC) a.k.a. MSC a.k.a. skeletal stem cells (SSC), and terminally differentiated stage, osteocytes (OT). Other types of cells important for bone homeostasis and repair, such as osteoclasts, are beyond the scope of this review for various reasons. For example, with regards to osteoclasts, we do not have enough data on their energy metabolism. Energy metabolism in osteoclast precursors, hematopoietic cells, is relatively well studied and has been reviewed elsewhere. Energy metabolism specifics in chondrogenic and adipogenic lineages are extensive enough to be standalone issues.

## 2 Cellular bioenergetic machinery

### 2.1 Glycolysis

Bioenergetic machinery of the cell represented by the glycolytic pathway and mitochondrial compartment, has been thoroughly described in numerous textbooks and reviews. Therefore, we will only briefly discuss the glycolytic and mitochondrial pathways and highlight the points that are frequently overlooked.

The glycolytic pathway converts six-carbon glucose to two three-carbon molecules of pyruvate in nine or ten steps. Each step of glycolysis is controlled by a specific enzyme. As a result, 2 ATPs and 2 NADH are produced per one molecule of glucose catabolized. There are several important points that are often overlooked: 1) Most steps of glycolysis are reversible and reactions can go in the opposite direction contributing to gluconeogenesis and other processes; 2) Glycolysis is tightly coupled with the Pentose Phosphate Pathway (PPP) and through it to NADPH production, lypogenesis, and nucleotide synthesis (Moffatt and Ashihara, 2002), and with glycogenesis; 3) Relevant to the previous point, glycolytic activity keeps cells in a reduced state because PPP-derived NADPH is a major redox regulator; 4) The glycolytic process while relatively inefficient, is fast and therefore useful for cells undergoing rapid proliferation or extreme workloads (Zhu and Thompson, 2019); and 5) Glycolytic process cannot proceed if cytosolic NADH is not converted to NAD^+^ via either pyruvate fermentation into lactate by Lactate Dehydrogenase (LDH) or mitochondrial oxidative pathway coupled with the malate-aspartate shuttle (MAS) activity.

### 2.2 Mitochondria

Mitochondria are double-membraned organelles containing circular mitochondrial DNA (mtDNA), multiple enzymes of the TCA cycle, components of the electron transport chain (ETC), and numerous transporters. These organelles can uptake glucose-derived pyruvate as well as glutamine, fatty acids, and other fuels for the process of oxidative phosphorylation (OxPhos). As a result of OxPhos, up to 36 ATPs are produced. Mitochondria are also a major source of cellular ROS, biosynthetic hubs performing various steps of lipid and amino acid biosynthesis, regulators of cellular calcium signaling, and producers of substrates for epigenetic reactions, such as acetyl-coenzyme A (AcCoA), α-ketoglutarate, and 2-hydroxyglutarate. Important points to remember are: 1) Mitochondrial outer membrane (OMM) is relatively permeable while the inner mitochondrial membrane (IMM) is impermeable to most ions and compounds unless specific transporters are present. IMM integrity is crucial for mitochondrial OxPhos and other activities (Brand and Nicholls, 2011); 2) Mitochondria can fuse and fission depending on cell requirements and can exchange mtDNA, proteins, and metabolites during fusion. Fission is required for normal cell division, autophagic mitochondrial quality control, and execution of apoptosis (Cook et al., 2017; Civenni et al., 2019); 3) While showing high degree of autonomy, mitochondria interact with and depend on the cytoskeleton, endoplasmic reticulum, and other cellular structures; 4) Many mitochondrial enzymatic reactions are reversible and can go in either direction which is important for example for the processes of reductive carboxylation and lipid biosynthesis (Mullen et al., 2011; Mullen et al., 2014; Nowinski et al., 2020); 5) mtDNA has limited ability to repair and, thus, much more vulnerable to damage when compared to nuclear DNA (Picard et al., 2016; Kopinski et al., 2021; Singh et al., 2021); 6) More active mitochondria are more involved in the execution of apoptosis and, therefore, mitochondrial oxidative metabolism sensitizes cells to cell death (Whelan et al., 2012; Ferranti et al., 2020); and 7) Opening of a large non-selective Mitochondrial Permeability Transition Pore (MPTP) controlled by cyclophilin D (CypD) is a convergence point for various pathogenic signals leading to loss of IMM integrity and mitochondrial dysfunction (Giorgio et al., 2010; Bernardi et al., 2015a; Bernardi et al., 2015b; Bernardi et al., 2021).

## 3 Bone and bone marrow niche: availability of oxygen and bioenergetic substrates

### 3.1 Oxygen

**Fig. 1:**
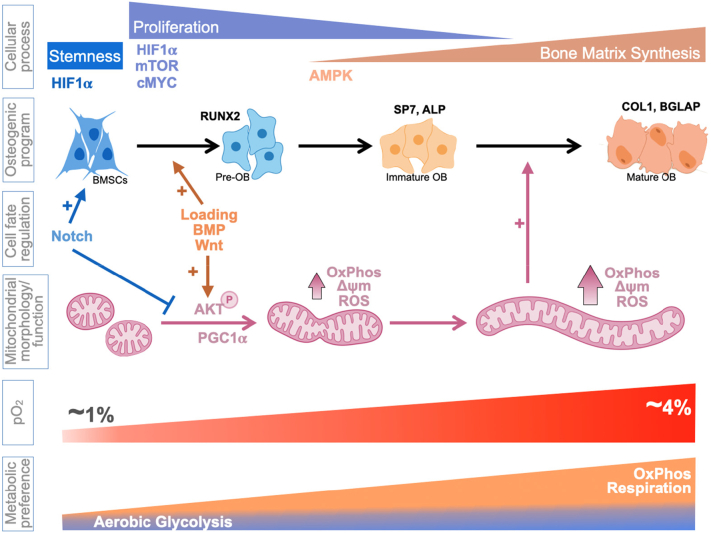
Bioenergetic regulation and signaling during osteoblast differentiation.

Historically, bone and bone marrow have been considered hypoxic. This was based mostly on indirect measurements, such as the levels of HIF and HIF-dependent markers. This view has affected the field's take on energy metabolism in bone-resident cells and, in particular, in osteoblasts and osteocytes. If the microenvironment is hypoxic, cells have to adjust by relying less on oxygen-dependent mitochondrial process of OxPhos and more on glycolytic fermentation, i.e. showing the Pasteur effect (Barker et al., 1964). There are three counterarguments to this line of thinking. First, with the development of new technology-based methods of measuring oxygenation and vascularization in vivo, we now have more accurate data on the oxygen levels within the bone and at various distances from the bone surface in the bone marrow. We also have a more detailed picture of bone and bone marrow vascular networks including capillaries. These new data indicate that while bone marrow is for the most part indeed hypoxic, bone is much better oxygenated then was considered before. Bone marrow shows the average pO_2_ of ~1.8% with the range of 0.6–2.8% depending on the distance from the vessel (Spencer et al., 2014). Cortical bone, however, is better oxygenated with pO_2_ close to 4% within the bone and ~2% at the endocortical surface. This value is progressively decreasing with increasing distance from the endocortical surface reaching ~1% at 40 μm, i.e. 4–6 cell layers from the bone. Since trabecular bone is vascularized presumably as well as cortical bone and oxygen comes from bone vessels rather than from bone marrow vasculature, oxygenation of trabecular bone and endosteal surfaces is likely similar to the cortical bone values. Based on the above data, we may conclude that while BMSCs likely reside in a hypoxic environment within the bone marrow, osteoblasts and osteocytes have adequate oxygen supply (Fig. 1). The reason for low pO_2_ values in highly vascularized bone marrow is still unclear and requires further investigation. We can only speculate that it could be due to known higher permeability or ‘leakiness’ of bone marrow vessels (Passaro et al., 2017) when compared to other tissues and high cellularity of bone marrow so that the consumption greatly exceeds supply. Poor oxygen diffusion may also play a role as it is known that oxygen level decreases by ~90% at a distance from the vessel equivalent to 10 cell diameters (Gatenby et al., 2007). For comparison, glucose drops only by ~10% at the same distance.

The second counterargument has to do with the issue of how ‘hypoxic’ is bone and bone marrow hypoxia? In other words, is this level of hypoxia severe enough to inhibit OxPhos? While bone marrow pO_2_ of 0.6–2.8% can stabilize and, therefore, activate HIF which promotes the glycolytic process, it is not severe enough to directly affect mitochondrial ETC. Oxygen is the final electron acceptor of the mitochondrial ETC where it is consumed at the cytochrome *c* oxidase (COX) of respiratory complex IV. Therefore, oxygen deficiency effectively puts a brake on the ETC activity. However, the estimated *Km*(O_2_) of COX is ~1 μM (Krab et al., 2011) which corresponds to pO_2_ of ~0.6 mmHg or ~0.08%. Therefore, even the lowest pO_2_ values found in the bone marrow (0.6%) are not severe enough to directly lower the activity of the ETC in the bone marrow resident cells. ETC in osteoblasts residing on the bone surface with pO_2_ of 2% is even less likely to be inhibited by low oxygen. HIFs are expected to be activated in these cells and may exert some inhibitory effects on OxPhos. Osteocytes, residing at pO_2_ of 4% are not expected to be affected even by HIF. In sum, hypoxia is unlikely to be a limiting factor for OxPhos activity in osteoblasts and osteocytes but may be a limiting factor for OxPhos activity in bone marrow BMSCs due to HIF presence.

The third counterargument is that HIF1 can be activated independently of pO_2_ by various means, such as high Akt and mTOR activity, ROS, accumulation of succinate due to blockade of TCA cycle, etc. Such conditions can be observed not only in pathology but also in some physiological states, i.e. during rapid proliferation or when cells suppress OxPhos to maintain a reduced state favoring cell ‘stemness’. We, in fact detected high HIF1 activity in undifferentiated BMSCs incubated at room pO_2_ of 21% (Shum et al., 2016a). Therefore, elevated HIF1 levels do not always indicate hypoxia. This is a very important observation when analyzing and drawing conclusions from non-functional data such as transcriptomic data.

### 3.2 Bioenergetic substrates

With regards to availability of bioenergetic substrates transported by the blood, we do not envision any deficits for any cell compartments within bone because of high vascularization. Recent studies indicate that even dense cortical bone contains a rich network of capillaries likely reaching osteocytes buried deep inside the bone (Gruneboom et al., 2019). Moreover, nutrients, such as glucose, are known to diffuse significantly longer distances from the vessel than oxygen (Gatenby et al., 2007). Therefore, even cells deep within the bone marrow stroma and far from vessels are anticipated to have adequate nutrient supply. Glucose uptake by the bone has been measured in vivo in mice using [^18^F]-Fluorodeoxyglucose (FDG) and positron emission tomography (PET) scan (Zoch et al., 2016). They observed quite significant glucose uptake by various bones in mature animals comparable to that observed in the liver but significantly lower than that in the lung or spleen. Another major fuel for cells is glutamine, the most abundant amino acid in the blood (Roth, 2008). Glutamine has been in fact shown to be as important for bone tissue as glucose (Yu et al., 2019). Bone tissue is also capable of taking up significant amounts of lipids (Kim et al., 2017). In addition, bone marrow contains marrow adipose tissue (MAT), a valuable local source of highly energetic triglycerides. Recent studies indicate that MAT in fact plays an important role in providing bioenergetic fuel for osteogenic lineage cells (van Gastel et al., 2020).

## 4 Regulation of bioenergetics as relevant to bone and osteogenic lineage

In this section, we will discuss how several key signaling pathways physiologically relevant to osteogenic lineage cells, affect cell bioenergetics.

### 4.1 HIF

As was discussed above, bone marrow and bone surfaces facing bone marrow have pO_2_ levels (0.6–2.8%) that are sufficiently low to stabilize HIF. Therefore, it is important to understand how HIF influences bioenergetics in osteogenic lineage cells. HIF1α and HIF2α have been shown to play important roles in osteogenic lineage, showing both stimulatory and inhibitory effects depending on the context (Bentovim et al., 2012; Lee et al., 2021). With regards to bioenergetics, both HIF isoforms exert mostly inhibitory effect on OxPhos and stimulatory effect on glycolysis (Fig. 1). Both HIFs induce glucose uptake and PDK expression leading to inhibition of pyruvate oxidation (Kierans and Taylor, 2021). HIF1α also induces expression of several glycolytic enzymes including LDHA, which contributes most to diverting pyruvate from oxidation to fermentation into lactate (Singh et al., 2017). HIF2α is also known to inhibit β-oxidation of fatty acids (Liu et al., 2014). Therefore, opposite effects of HIFs on osteogenic lineage may be explained by their opposite effect on glycolysis and OxPhos and by the differentiation status of the affected cells. Stem cells and activated proliferating cells are known to thrive in a hypoxic, high HIF level environment. Thus, HIF gain- or loss-of-function may have positive or negative effect on cells at these stages, respectively. The effects are likely opposite in cells that rely more on OxPhos. Additionally, it is important to have in mind that some post-translational modifications require O_2_ as a cofactor, e.g. proline and collagen hydroxylation during matrix biosynthesis and α-ketoglutarate-dependent demethylation reactions during epigenetic regulation. Therefore, HIF can be stabilized to slow down TCA cycle activity and divert some O_2_ from OxPhos use to protein hydroxylation when pO_2_ levels are not sufficient to feed both processes.

### 4.2 Notch

Notch signaling is an important regulator of stemness in various embryonic and somatic stem and progenitor cells including BMSCs. It involves ligation of a receptor on the plasma membrane, release of the Notch intracellular domain (NICD), NICD nuclear translocation and interaction with RBPJκ and other factors, and expression of *Hes, Hey* and other target genes. The role of Notch in cell bioenergetics can be attributed primarily to its crosstalk with HIF1α. NICD has been shown to interact with HIF1α in the nucleus and potentiate its gene regulatory function (Gustafsson et al., 2005). Notch also induces STAT3 signaling needed for HIF1α expression (Moriyama et al., 2018) leading to upregulation of a variety of genes encoding glycolytic enzymes. Therefore, with regards to bioenergetics in BMSCs and osteogenic lineage, Notch signaling appears to be a pro-glycolytic regulator. However, one recent report by Lee and Long (Lee and Long, 2018) demonstrated that abnormally increased Notch signaling suppresses not only mitochondrial but also glycolytic metabolism.

### 4.3 Wnt

Wnt/β-catenin is a key osteogenic differentiation signaling pathway. It promotes activation of BMSCs, proliferation, and early osteogenic differentiation steps. Wnt signaling has been shown to exert somewhat conflicting effects on bioenergetics in osteogenic cells. One early report by An et al. (2010) showed activation of OxPhos by 50 ng/ml of Wnt3a in C3H10T1/2 cells. This effect was due to β-catenin-dependent expression of PGC-1α, a key inducer of mitochondrial biogenesis. The glycolytic activity was not measured in that work. Later, Esen et al. (2013) reported that 50 ng/ml Wnt3a mostly activated glucose uptake and glycolytic lactate production in ST2 cells with no visible effect on OxPhos. The effect was not mediated by canonical β-catenin but by the mTORC2-Akt signaling. Our group has recently published work showing that 25 ng/ml Wnt3a rapidly induced OxPhos in both ST2 and MC3T3 cells by activating Akt which phosphorylated multiple targets in mitochondria (Smith and Eliseev, 2021). There was also a less pronounced stimulatory effect on glycolysis in ST2 cells consistent with the earlier observation by Esen et al. Thus, Wnt signaling likely stimulates both glycolysis and OxPhos and its effect depends on the dose and duration of the treatment. One weakness in all these studies is the use of cell lines rather than primary cells because the process of immortalization is known to affect metabolic programming (Zhu and Thompson, 2019). We are also missing a comprehensive study of how manipulation of Wnt signaling in vivo affects bioenergetics in osteogenic lineage cells and bone tissue. Even though there is a clear correlation between the decline in Wnt/β-catenin signaling and bioenergetic changes observed in aging (Section 7.2), such an association is only speculative and needs to be further studied.

### 4.4 BMP

BMP signaling is central to osteogenic differentiation. BMP-dependent Smad1/5/8 transcription factors regulate a variety of osteoblast-specific genes, such as *Runx2* and others (Massague et al., 2005; Wang et al., 2014). BMPs also signal via non-canonical pathway by acting on Akt, mTOR, and MAPK. The effect of BMPs on cell bioenergetics in osteogenic lineage is still being investigated. In the paper described in the previous subsection, in addition to Wnt3a, Esen et al. investigated the effect of 300 ng/ml BMP2 on bioenergetics in ST2 (Esen et al., 2013). They did not observe any increases in the glucose uptake or glycolytic lactate production while the effect on OxPhos was not assessed. In our recent work, we investigated the effect of 25 ng/ml BMP2 on bioenergetics in osteogenic ST2 and MC3T3 cells and observed rapid induction of OxPhos with marginal effect on glycolysis (Smith and Eliseev, 2021) in both cell lines. Similarly to the effect of Wnt3a, these effects of BMP2 were mediated via Akt and Akt-mediated phosphorylation of various mitochondrial targets. BMPs were also reported to activate mTOR (Karner et al., 2017) and HIF1α (Huang et al., 2020; Huang et al., 2021). Since both mTOR and HIF1α stimulate glycolysis, we may anticipate some stimulatory effect of BMPs on glycolytic metabolism in osteogenic lineage. However, mTOR is also known to activate glutamine metabolism in mitochondria (Csibi et al., 2013), an oxidative process. Finally, it should be noted that the same comments apply here as in the above subsection describing Wnt effects on bioenergetics: absence of data on primary cells and in the in vivo settings.

### 4.5 PTH

Parathyroid hormone (PTH) is an important anabolic factor regulating osteogenesis when administered intermittently rather than continuously. PTH has also been studied with regards to its effect on cell bioenergetics in osteogenic lineage. Esen et al. reported (Esen et al., 2015) that 48 h treatment with 250 ng/ml PTH(1-34) increased glucose uptake and both glycolytic lactate production and OxPhos in MC3T3 cells by inducing Igf1/mTOR signaling. We used significantly lower dose of PTH(1-34) at 1 nM (4.1 ng/ml) based on the literature showing (Maeda et al., 2013; Ricarte et al., 2018) stimulation of downstream signaling at that dose (Esen et al., 2015). After 12 h incubation, we did not detect any significant effects on either OxPhos or glycolysis in both ST2 and MC3T3 cells (Smith and Eliseev, 2021). However, in our unpublished studies, we observed induction of glycolysis by 1 nM PTH (1-34) in primary human BMSCs after 24 h of treatment (146 ± 21% vs control, *P* = 0.02 by *t*-test, *n* = 5) and inhibition of OxPhos which did not reach significance. There is also a large body of literature showing inhibitory effect of PTH on both glycolysis and OxPhos but all of those studies were done in tissues other than bone and used excessive doses relevant to hyperparathyroidism (Rasmussen and Ogata, 1966; Rasmussen et al., 1967; Baczynski et al., 1985). Overall, most data point to a stimulatory effect of physiologically relevant doses of PTH on glycolysis in osteogenic lineage cells. This is logical since the effect of PTH is usually associated with the commitment, activation, and proliferation stages of osteoblast differentiation. As was mentioned above, glycolysis is the preferred means of energy production during these processes.

### 4.6 Mechanical stimulation

BMSCs and osteogenic lineage cells are mechanosensitive. They express mechanoreceptors, such as Piezo 1 and 2 and TRPV (Thompson et al., 2012; Sugimoto et al., 2017), and respond to mechanical load by activating various signaling pathways (Kido et al., 2010; Rath et al., 2011; Song et al., 2017; Yu et al., 2017a; Yu et al., 2018). Mechanical stress is an important element in bone formation, growth, and maintenance (Robling et al., 2006; Malinski et al., 2020). Cellular mechanosensitive ion channel activation has been shown to lead to higher expression of osteogenic markers, increased osteoblast differentiation, and bone formation (Song et al., 2017; Robling et al., 2006; Moss, 1997). Conversely, unloading inhibits osteogenic signaling, diminishes OB differentiation, triggers inflammatory markers, and can increase pre-osteoclast recruitment and activation leading to increased bone resorption (Robling et al., 2006). This process is observed in disuse models, such as hindlimb suspension and suggested as a causal factor for the onset of osteoporosis (Lieberman, 2016). One aspect that is frequently overlooked is that mechanical stimulation is a strong modulator of cell bioenergetics.

Although the direct link between mechanical stress and mitochondrial function has yet to be established, fluid shear stress in osteogenic cells activates Ca^2+^ transients which increases mitochondrial Ca^2+^ uptake needed for TCA cycle activity. Therefore, mechanical load effect on osteogenic cells rely on mitochondrial bioenergetic state (Yu et al., 2018). In vivo models, revealed that activation of SIRT1/3 significantly increases bone mass in the hind limb unloading model (Farr and Almeida, 2018). Mitochondrial Sirtuin 3 supports OxPhos and increases PGC-1α expression, a key regulator in mitochondrial biogenesis. SIRT3 also mediates deacetylation of CypD and thereby decrease in MPTP activity and increase in OxPhos. OBs from mechanically loaded bones presented significant changes in genes regulating metabolic pathways and the ERK1/2 MAPK signaling, an important regulator of mitochondrial dynamics (Galea et al., 2017). In contrast, microgravity exposure, a proxy of unloading, leads to mitochondrial dysfunction in humans and mice, presenting a metabolomic signature consistent with premature aging and oxidative stress (da Silveira et al., 2020). In vitro studies revealed important changes in genes regulating glucose uptake and oxygen consumption in osteocytes subjected to microgravity (Uda et al., 2021). Metabolomic and proteomic analysis showed that microgravity changes respirasomes' mass and decreases ATP synthesis along with triggering mitochondrial dysfunction and oxidative stress, ultimately leading to a decrease in OB function (Michaletti et al., 2017).

The diagram in Fig. 1 summarizes metabolic niche and regulation of bioenergetics in osteolineage cells.

## 5 Crosstalk of bone cell bioenergetics with other cellular and organismal functions

Glycolytic and mitochondrial pathways are well integrated into various cellular processes and play important regulatory role in these processes as discussed below.

### 5.1 Signaling

Cell signaling involves the initiation, processing, and transduction of signals as well as the response to such signals. The interplay of cell energy metabolism and cell signaling is a well-known phenomenon. Cells must respond quickly to various challenges redirecting metabolic pathways, modulating cell signaling, and adapting to environmental demands.

Osteogenic lineage cells possess sophisticated metabolite- and energy-sensing mechanisms, such as AMPK and mTOR (Huang et al., 2015; Li et al., 2018). AMPK signaling senses glucose levels and energy charge whereas mTOR is the major mediator of amino acid sensing. AMPK via PGC-1α induces mitochondrial biogenesis and thus OxPhos (Fernandez-Marcos and Auwerx, 2011). AMPK can also stimulate BMP2 expression (Jeyabalan et al., 2012; Ahier et al., 2018), which in turn induces OxPhos as was recently shown by our group (Smith and Eliseev, 2021). On the other hand, AMPK is inhibited by mTOR pathway (Gonzalez et al., 2020; Ling et al., 2020). MTOR in osteogenic lineage is important for preosteoblast proliferation (Fitter et al., 2017) while its inactivation is required for OB terminal differentiation and maturation (Huang et al., 2015). With regards to the effect on bioenergetics, mTOR pathway promotes glycolysis by inducing HIF1α and c-Myc (Linke et al., 2017). HIF1α effect on cell metabolism has been discussed above. C-Myc is an important transcription factor activator of pro-proliferative genes during skeletal development (Zhou et al., 2011). Interestingly, c-Myc is transcriptionally repressed by BMP/Smad signaling (Massague et al., 2005). Given that energy-sensing AMPK contributes to BMP/Smad activation, it is reasonable to suggest that the end of OB proliferative stage is accompanied by a metabolic shift. Additionally, BMP/Smad-induced RUNX2 was shown to increase *Glut1* expression in preosteoblasts (Wei et al., 2015). Upregulation of *Glut1* supports glycolysis needed for proliferation and for supplying the TCA cycle to sustain the OxPhos-dependent steps of OB differentiation.

Calcium is an important second messenger involved in a variety of signaling pathways (Carafoli and Krebs, 2016). Ca^+2^/Calmodulin-dependent kinase II (CaMKII) cascade is described to regulate OB differentiation by increasing Osterix protein levels and transcriptional activity (Choi et al., 2013). Calcium influx can activate the calcineurin-NFAT pathway effecting downstream Wnt signaling and therefore, supporting OB differentiation (Fromigue et al., 2010). Spatial and temporal intracellular calcium control is also shown to play a role in OB differentiation regulating the activity of calcium-mediated stress kinase p38 MAPK which coordinates the expression of osteogenic genes (Eapen et al., 2010).

Mitochondria are known to be involved in calcium buffering and thus, in regulation of cytosolic calcium involvement in signaling (Gunter et al., 2000; Gunter et al., 2004). Additionally, Ca^2+^ is an important metabolic mediator within mitochondria. Cytosolic Ca^2+^ activates the adenine nucleotide translocase whereas intramitochondrial free Ca^2+^ mediates the rate of OxPhos and activates the electron transport chain (ETC) and F_1_F_0_ ATP synthase (Gunter et al., 2004). Intramitochondrial Ca^2+^ controls the activity of Ca^2+^-sensitive enzymes such as pyruvate dehydrogenase (PDH), isocitrate dehydrogenase (ICDH), and α-ketoglutarate dehydrogenase (α-KGDH). Therefore, the rate of NADH production and TCA activity is regulated by calcium influx into the mitochondria. ETC and TCA activation support a high mitochondrial membrane potential (ΔΨm) which is regarded as the main driving force for mitochondrial Ca^2+^ uptake (Gunter et al., 2000). Such coordination and interconnection of events support the idea that proper OB function and bone formation, which are highly dependent on calcium signaling and transport, is concomitant with OxPhos activation and mitochondrial function.

We have previously demonstrated that osteogenic signals, such as BMP2 or Wnt3a, induce OxPhos via Akt signaling (Smith and Eliseev, 2021). Akt signaling axis, another crucial energy sensor, is interconnected with AMPK and mTOR pathways. Akt has been shown to regulate cellular metabolism by preventing mTORC1 complex formation and stimulating mitochondrial function and formation of mitochondria-associated membranes (Betz et al., 2013). An interesting new direction has recently been developed connecting Akt, mitochondria, and microRNA (miR). During osteogenic differentiation, certain miRs were shown to activate Akt and through it, mitochondrial metabolism (Zheng et al., 2019). Moreover, miRs were found to reside in mitochondria although their exact role there remains to be unveiled (Zheng et al., 2021).

A key feature in all signaling pathways described above is the metabolite/energy sensor mediation. Such sensors are the interface of the environment and biological processes connecting the metabolite-sensing and signaling. The sensor is responsible for perceiving the nutrient status of the environment and transforming this chemical kinesis into a cell signaling pathway. In turn, the signaling pathway can redirect or adjust cell metabolism to support cell fate decisions in coordination with metabolite availability, developmental stage, or stress status of the cell. Not surprisingly, signaling proteins can be conjugated with sugar, lipid, amino acid, and metabolic intermediates. This conjugation allows a non-enzymatic or more frequently, enzymatic transfer of the metabolite to further transmit the signal, either with a metabolic or transcriptional effect (Wang and Lei, 2018). There is a vast list of protein post-translational modifications depicting this important role of metabolite association with proteins. A great example of metabolite-sensing rewiring of cellular metabolism is the exposure of osteoprogenitor cells to galactose instead of glucose (Shares et al., 2018). Since galactose yields no net ATP during glycolysis, cells activate OxPhos to make ATP in the presence of mitochondrial substrates. We showed that OxPhos activation in such scenario changes osteogenic program. Citrate derived from AcCoA in active mitochondria is translocated to the cytosol, converted back to AcCoA by ATP-citrate lyase (ACLY) enzyme, and participates in ACLY-dependent acetylation and activation of β-catenin, stimulating downstream osteogenic program (Shares et al., 2018).

In sum, signaling can affect cell metabolism and vice versa, thereby influencing cell fate decisions in osteogenic lineage.

### 5.2 Biosynthesis

We can divide cellular biosynthesis into two main types: 1) biomass accrual during cellular proliferation, which is discussed in the Proliferation subsection; and 2) specialized biosynthesis that is characteristic of certain cell types according to their role and function, as discussed in this subsection.

Highly specialized cells are shown to require OxPhos to function properly. Specialized biosynthesis requires metabolite precursors and intermediates either as building-blocks or as signal transducers. OB function involves extracellular matrix deposition and mineralization. Approximately 90% of bone organic matrix is collagen type I. Collagen I synthesis and maturation are complex processes. The self-assembly of pro-chains in a triple helix is preceded by proline and lysine hydroxylation, which requires oxygen, iron, and ascorbate as cofactors and α-ketoglutarate as a reducing agent (Viguet-Carrin et al., 2006). Oxidative pathway is a prevalent source of α-ketoglutarate. Biochemically, collagen is approximately one third glycine, the most prevalent, and one fourth proline, the second most prevalent amino acid (Szoka et al., 2015; Albaugh et al., 2017). Other amino acids such as glutamine, glutamate, arginine, and ornithine can be interconverted to proline. Since active mitochondria participate in glutamine conversion to glutamate and in urea cycle (Albaugh et al., 2017), proline synthesis is coupled to energy metabolism via OxPhos. In addition to de novo synthesis, collagen recycling plays an important role in collagen synthesis. Imidodipeptides derived from collagen degradation, can be used for collagen re-synthesis. The hydrolysis of imidodipeptides is catalyzed by the cytosolic enzyme prolidase. Decreased prolidase activity can be a step-limiting factor in collagen biosynthesis. Interestingly, a glycolytic intermediate, phosphoenolpyruvate, is a major inhibitor of prolidase activity (Szoka et al., 2015) suggesting that high rates of glycolysis may impair collagen recycling and thus biosynthesis. Collectively, the above data point to the supporting role of OxPhos in collagen I synthesis and maturation in OBs.

### 5.3 Epigenetics

Epigenetic modifications are highly dynamic and constantly reshape chromatin landscape during development and homeostasis. Such a remodeling provides chromatin the necessary plasticity to modulate cell fate decisions in response to specific environmental or metabolic changes. Removal of methyl marks from DNA and histones are important post-translational modifications (PTM) regulating the chromatin structure and therefore, playing a role in gene expression, cellular differentiation and lineage specification (Reid et al., 2017; Gao et al., 2018). α-KG is a crucial co-factor for demethylase enzymes, such as the α-KG-dependent histone lysine demethylases, including Jumonji family, and α-KG-dependent TET family deoxygenases that catalyze DNA cytosine demethylation (An et al., 2017; Tran et al., 2019; Baksh and Finley, 2021). Mitochondria are the most abundant source of α-KG within the cell, utilizing both glucose and glutamine to sustain high levels of α-KG when required. On the other hand, 2-hydroxyglutarate (2HG) is a competitive inhibitor of multiple α-KG-dependent deoxygenases and is shown to prevent cellular differentiation by inducing chromatin hypermethylation. Interestingly, 2HG is also produced in mitochondria under pathological conditions, such as IDH enzyme mutations or abnormally high reductive carboxylation as seen during hypoxia when the enantiomer L-2HG can arise from reduction of glutamine-derived α-KG (Intlekofer et al., 2015). An intermediate from glycolysis, 2/3-phosphoglycerate, can be directed into the one-carbon cycle, ultimately producing S-adenosyl methionine (SAM), which is used as a methyl donor for histone and DNA methylation reactions (Reid et al., 2017; Schvartzman et al., 2018). Although there is no clear correlation between overall methylation level and differentiation, some osteogenic genes such as *BGLAP*, are reported to be in a hypomethylation state in OBs when compared to undifferentiated cells (Yu et al., 2017b). This suggests an important control of DNA demethylation-mediated regulatory mechanism during osteoblast differentiation.

Histone acetylation is also known to play an important role in cell fate by maintaining chromatin in an opened state, which can either sustain pluripotency or induce cellular differentiation favoring lineage-specific TF binding (Ryall et al., 2015; Atlasi and Stunnenberg, 2017). Acetyltransferases use AcCoA as a substrate for acetylation. AcCoA, generated mostly through the mitochondrial oxidation of pyruvate, is converted to citrate. Citrate, in turn, can exit mitochondria and be converted back to AcCoA by cytosolic ACLY (Shares et al., 2018). On the other hand, the nuclear ACLY enzyme can convert citrate transported extracellularly though citrate transporters in the plasma membrane and then into the nucleus into AcCoA. The pyruvate dehydrogenase complex can also translocate into the nucleus and use glycolysis-derived pyruvate to generate high concentrations of AcCoA (Schvartzman et al., 2018). Therefore, high mitochondrial activity cannot be exclusively associated with elevated levels to histone acetylation.

Our current knowledge demonstrates that bioenergetics plays an important regulatory role in chromatin modifications and, consequently in cellular differentiation. However, it is unclear how a specific metabolic pathway would favor a particular histone or DNA modification during cell differentiation. Since chromatin modifications are carried out by enzymatic reactions, several factors can influence the allosteric regulation and enzymatic rate of a reaction. Therefore, whether substrate concentrations or product levels are limiting or favoring factors to enable proper enzymatic activity regulating chromatin modifications is still debatable (Schvartzman et al., 2018). Additionally, some enzymes can translocate to the nucleus to mediate local nuclear production of metabolites to fuel chromatin modifications, as examples presented above.

### 5.4 Proliferation

The process of OB differentiation involves a proliferative phase during pre-osteoblast commitment. As OB maturation takes place, proliferation is halted, and high rates of matrix synthesis and deposition are observed. Cell proliferation requires the accumulation of intracellular biomass sustained by macromolecule biosynthesis which is fed mostly by glucose. Among such macromolecules are nucleotides, necessary for DNA replication, and proteins and lipids that enter the composition of structural and functional cellular systems such as the cytoskeleton, organelles and biological membranes (Zhu and Thompson, 2019; Palm and Thompson, 2017).

The endogenous nucleotide generation is executed by the pentose phosphate pathway (PPP) branched from the glycolytic pathway. The PPP starts with glucose-6-phosphate dehydrogenase (G6PD) converting glucose-6-phosphate into ribose-5-phosphate used for nucleotide production, mostly for purine biosynthesis. However, the final assembling of purine nucleotides is dependent on metabolites and intermediates coupled to TCA cycle (Moffatt and Ashihara, 2002). Pyrimidine biosynthesis is also coupled to TCA cycle. Glutamine is the major amide group donor in pyrimidine synthesis (Zhu and Thompson, 2019). Interestingly, dihydroorotate dehydrogenase, a key enzyme in pyrimidine pathway localized in the inner mitochondrial membrane, harvests electrons from ubiquinone and transfers them to complex III. Therefore, pyrimidine biosynthesis is directly coupled to electron transport chain and mitochondrial function.

The increase in glycolytic flux seen in proliferative cells also supports fatty acid and amino acid synthesis, including non-essential amino acids. Not surprisingly, mTORC activation is fundamental to sustain cell proliferation by either regulating de novo amino acid synthesis and pyrimidine production. AcCoA is the major intermediate utilized for fatty acid synthesis, it precedes malonyl-CoA conversion and later elongation. Although glycolysis is the main carbon source for proliferating cells, glutamine uptake is also present, as described above, and important for anaplerosis. Reductive carboxylation of glutamine can feed into the fatty acid biosynthesis (Palm and Thompson, 2017).

In sum, glycolysis appears to be a major bioenergetic pathway supporting proliferation, however some level of mitochondrial activity is also necessary.

### 5.5 Differentiation

Differentiated somatic cells tend to use OxPhos as preferred pathway for energy production, and therefore produce more ROS leading cells to a higher oxidative state. Conversely, undifferentiated cells are shown to rely on glycolysis for ATP generation (Dahan et al., 2019). By favoring glycolysis, undifferentiated cells can preserve low levels of ROS, maintain a reduced state, prevent genomic and mitochondrial DNA damage, and lipid and protein oxidation. A reduced status is shown to promote self-renewing division along with increased levels of protein O-GlcNAcylation. Sustained by high levels of glycolytic flux, O-GlcNAcylation can maintain stemness and pluripotency by regulating signaling pathways, chromatin structure, and organelle dynamics. Conversely, a decrease in O-GlcNAcylation levels is shown to disrupt self-renewal and reprogramming pushing cell towards differentiation (Dahan et al., 2019). Additionally, undifferentiated cells contain mostly fragmented mitochondria with immature cristae, decreased expression of MFN1/2 and increased expression of uncoupling protein 2 (UCP2) (Dahan et al., 2019; Lisowski et al., 2018).

Differentiation exerts a metabolic shift presenting an early increase in lactate production, higher oxygen consumption rate and mitochondrial fusion with enlarged networking and cristae number. In fact, we have demonstrated such effects during OB commitment (Shum et al., 2016a). Differentiation, in general, is also shown to increase mitochondrial pyruvate carriers expression and decrease UCP2 expression, favoring pyruvate oxidation and OxPhos function (Dahan et al., 2019). OxPhos activation clearly increases the oxidative status within the cell through increased ROS generation, which is shown to promote cell cycle exit and differentiation (Noble et al., 2015). ROS oxidize cysteine residues of MAPK phosphatases, known to play an important role in osteoblast differentiation (Mandal et al., 2011). Not surprisingly, studies manipulating cellular redox balance have shown that more reduced cells undergo self-renewal and maintain stemness while more oxidized cells undergo differentiation (Noble et al., 2015). Additionally, as discussed above, oxidative pathway promotes acetylation of various proteins including β-catenin, RUNX2, and Osterix, leading to their activation and progression of OB differentiation. Therefore, the majority of data indicates that undifferentiated cells prefer low while more differentiated cells high mitochondrial OxPhos activity.

### 5.6 Overall energy balance

The bone tissue itself is an important regulator of the organism energy balance (Lee et al., 2007). Mature OBs deposit osteocalcin into the bone matrix. The carboxylated, or inactive, form of osteocalcin is an important building block of bone structure linking collagens to the mineralized matrix. The uncarboxylated active form of osteocalcin is formed upon osteoclast action on bone matrix and released into the blood stream (Moser and van der Eerden, 2018). Active osteocalcin functions as a hormone increasing cell metabolism. In the pancreas, osteocalcin acts on beta cells stimulating insulin release, and at the same time directing fat cells to release the hormone adiponectin, which increases sensitivity to insulin (Wei et al., 2014). Therefore, osteocalcin regulates glucose homeostasis. It is also shown to play a role in exercise capacity, brain development, and male fertility (Moser and van der Eerden, 2018). We showed that changes in mitochondrial function has a direct effect on bone tissue structure and osteocalcin content (Shum et al., 2016b; Shares et al., 2020). Thus, OB energy state is not only important for bone tissue but for the entire organism energy balance.

**Fig. 2:**
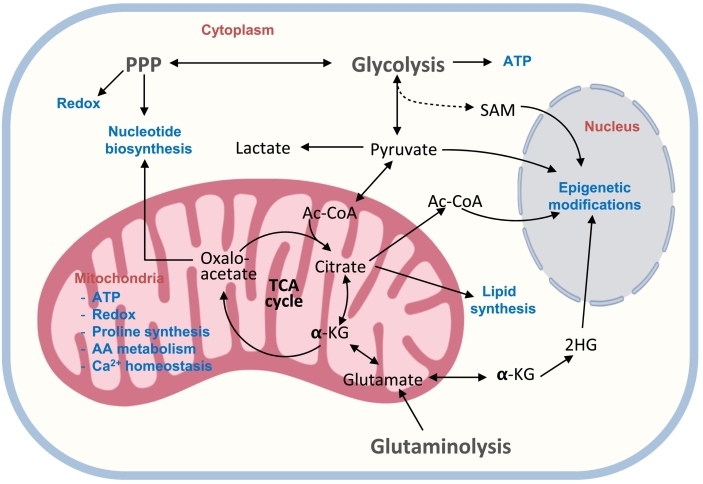
Regulation of cell functions by bioenergetic pathways. 2HG: 2-hydroxyglutarate, AA: amino acid, Ac-CoA: acetyl coenzyme A, α-KG: alfaketoglutarate, PPP: pentose phosphate pathway, SAM: S-adenosyl methionine, TCA: tricarboxylic acid.

The diagram in Fig. 2 a crosstalk between cell bioenergetics and various cellular functions.

## 6 The role of glycolytic and oxidative metabolism in osteogenic lineage

The most debated questions in the field are ‘what is the preferred method of energy production by osteoblasts, osteocytes, and their precursors, OxPhos or glycolysis?’ and ‘do osteoblasts have the Warburg effect, a.k.a. aerobic glycolysis?’ The concept of Warburg-like metabolic setup in bone cells is based on the early studies of bone metabolism showing that glucose is mostly metabolized into lactate and not via the TCA cycle (Borle et al., 1960; Cohn and Forscher, 1962). Those early studies were done either on primary cells digested from bone or on bone slices and used labeling techniques available at that time. While these early studies provided valuable information on bone metabolism, we now have a more complete picture of all the players involved as well as more accurate techniques. For example, we now know that glutamine and fatty acids are equally important for osteolineage cells so that these cells may not need glucose-derived pyruvate to feed into the TCA cycle (Nowinski et al., 2020; Yu et al., 2019; Kim et al., 2017). Also, the cell and tissue preparations used then, were not selective for osteolineage cells and likely included hematopoietic, blood, and other types of cells. Thus, the concept of Warburg-like metabolism in bone cells needs to be re-evaluated. We will start by discussing what the Warburg effect is. Most eukaryotic cells follow the Pasteur effect (Barker et al., 1964), i.e. they generate ATP primarily via OxPhos under normoxia and switch to glycolytic fermentation under hypoxia. However, many types of cancer cells continuously use glycolytic fermentation independently of oxygen concentration. Otto Warburg first described this metabolic abnormality in cancer cells and attributed it to some yet unknown defects in cell respiration machinery (Vaupel and Multhoff, 2021), i.e., as we now know, mitochondria. Warburg determined that in cancer cells showing aerobic glycolysis, more than 50% of total ATP is produced via glycolytic fermentation (Warburg, 1956). In normal differentiated cells, he found this value to be around 1% and in rapidly growing less differentiated cells, 20%.

We have analyzed recent literature data on osteogenic lineage. There is now a consensus that undifferentiated osteoprogenitors prefer glycolysis and show very low OxPhos activity (Shum et al., 2016a; Yu et al., 2019; Smith and Eliseev, 2021; Shares et al., 2018; Chen et al., 2008). This is consistent with the data on embryonic stem cells and somatic stem cells from other tissues (Shum et al., 2016a; Ryall et al., 2015; Dahan et al., 2019; Lisowski et al., 2018; Tsogtbaatar et al., 2020). Undifferentiated cells thrive in more reduced environment supported by glycolytic metabolism and low OxPhos (Dahan et al., 2019; Noble et al., 2015). Data on osteoblasts and osteocytes are less uniform. Table 1 gives a summary of what has been shown in the field in the last twenty years. With few exceptions, these studies detected activation of OxPhos during osteogenic differentiation of cells from different sources; so that osteoblasts (and likely osteocytes) can be categorized as actively respiring cells. Glycolytic activity in osteoblasts in most cases does not change when compared to undifferentiated cells. In addition to those in vitro data, we have recently received convincing in vivo evidence that more differentiated osteolineage cells show higher OxPhos. The study by Schilling et al. (2022) used two-photon (2P) microscopy and NAD(*P*)H fluorescence lifetime imaging (FLIM) to assess free vs bound forms of NAD(*P*)H reflecting glycolytic vs oxidative metabolism, respectively. Although NAD(*P*)H fluorescence has been utilized for in vivo metabolic studies before, 2P-FLIM of free vs bound NAD(*P*)H allowed for the first time to measure contribution of glycolytic vs oxidative metabolism in vivo. This approach clearly indicated that more differentiated cells, i.e. embedding OBs and OTs show activation of oxidative metabolism (Schilling et al., 2022).

**Table 1:**
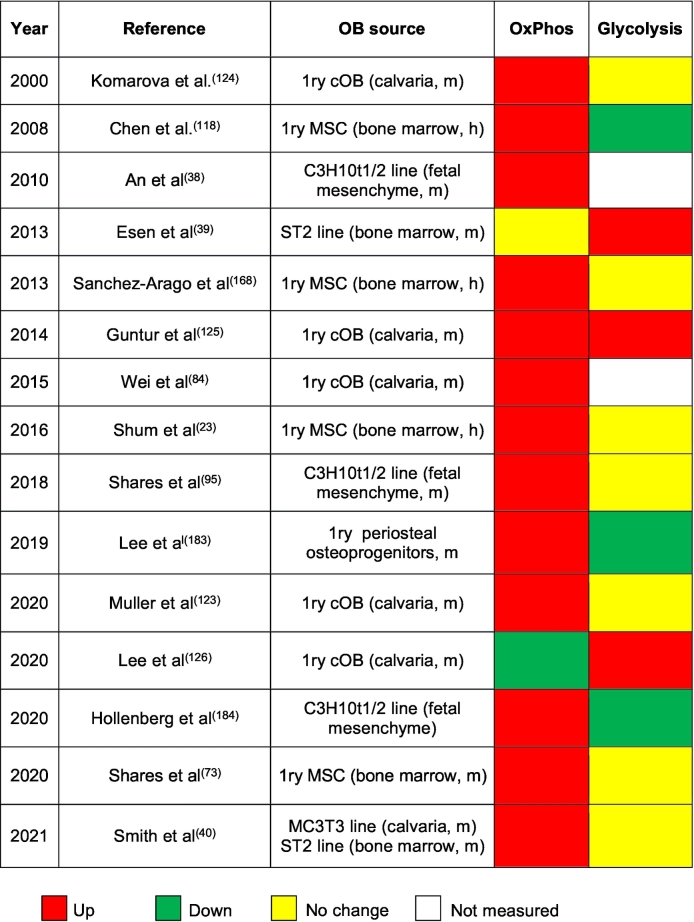
Changes in OxPhos and glycolytic activities during osteoblast differentiation. MSC, mesenchymal stem cells; cOB, calvarial osteoblasts; 1ry, primary; h, human; m, murine.

To determine whether the cell has the Warburg effect, i.e. it makes >50% of ATP via glycolysis in normoxia, one needs to trace glycolysis- vs OxPhos-derived ATP similarly to how it was done by Otto Warburg (Warburg, 1956). The modern method is to use the Seahorse bioenergetic profiler (Gu et al., 2021) to measure basal and maximal oxygen consumption rate (OCR), a measure of OxPhos, and extracellular acidification rate (ECAR) due to lactate production, a measure of glycolysis, and plot the so-called ‘energy-map’, i.e. OCR vs ECAR. We have used this method in our recent study which clearly showed that osteoinduction shifts cell metabolism towards the oxidative pathway (Smith and Eliseev, 2021). In addition, we have summarized OCR and ECAR values in osteoblasts from different studies and compared it to a cancer cell line of similar origin, osteosarcoma 143B, which has a well pronounced Warburg effect (Giang et al., 2013) (Table 2). Since these different studies used different units for OCR and ECAR and different normalization strategies, we recalculated these rates to express them all in nmoles O_2_/min/10^6^ cells and mpH/min/10^6^ cells, respectively. We also approximated that 10^6^ osteoblastic cells are equivalent to 0.2 mg of cell protein, the value we usually receive during protein extraction from OBs. Table 2 demonstrates that OBs from different sources show very different OCR and ECAR. With the exception of one study (Muller et al., 2020), data on OBs derived from primary bone marrow MSCs, show relatively low OCR and ECAR and, thus, overall metabolic rate. This likely reflects the fact that these cells are usually isolated from adult, skeletally mature animals or humans that have completed bone growth. The OCR to ECAR ratio in these cells is significantly higher than that of our Warburg effect reference 143B cells and, therefore, these bone marrow MSC-derived OBs do not have the Warburg effect. Immortalized cells from the same sources, human hFOB (Giang et al., 2013) and mouse osteoinduced ST2 (Esen et al., 2013; Smith and Eliseev, 2021), show higher OCR and ECAR than the primary cells (Table 2). This is not surprising, since these are transformed cells that are usually more active. However, the OCR to ECAR ratio is again significantly higher than that in 143B cells. Of note, both OCR and ECAR in ST2 cells in the study of Esen et al (Esen et al., 2013) are twice as high as in our study by Smith et al (Smith and Eliseev, 2021) but the OCR to ECAR ratio is similar. This indicates similar metabolic preferences in both studies even if the overall metabolic rate is different.

**Table 2:**
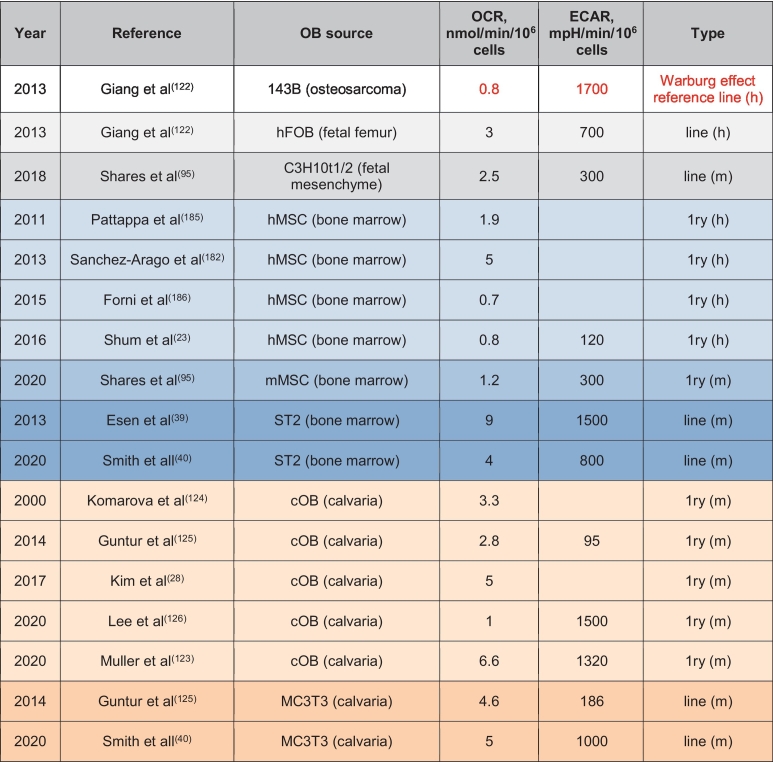
OCR and ECAR values in osteoblasts from different sources. MSC, mesenchymal stem cells; cOB, calvarial osteoblasts; 1ry, primary cells; h, human; m, murine.

Another widely used source of OBs for metabolic studies is primary calvarial cells (Kim et al., 2017; Muller et al., 2020; Komarova et al., 2000; Guntur et al., 2014; Lee et al., 2020) and calvaria-derived MC3T3 cell line (Smith and Eliseev, 2021; Guntur et al., 2014). These types of cells show the highest variability in both OCR and ECAR (Table 2). In osteoinduced primary calvarial cells, OCR ranges from 1 to 6.6 nmoles O_2_/min/10^6^ cells and ECAR from 95 to 1500 mpH/min/10^6^ cells. In osteoinduced MC3T3 cells, OCR is more uniform and is around 5 nmoles O_2_/min/10^6^ cells, while ECAR ranges from 186 to 1000 mpH/min/10^6^ cells. Not all of the above-mentioned studies evaluated both OxPhos and glycolysis but of the ones that did, only one recent study done in calvaria-derived OBs by Lee et al. (2020) showed OCR to ECAR ratio similar to that in 143B cells. Therefore, the Warburg-like metabolic setup was in fact observed in calvaria-derived OBs in that study. However, the majority of studies in OBs produce OCR to ECAR ratios not consistent with the Warburg phenotype (Table 2). There may be different reasons for the variability in reported OCR and ECAR values, such as the difference in calibration and sensitivities between individual devices, presence of supraphysiological levels of glucose and other fuels in the media, cell culture passage number, purity of cell preparation, and primary cell isolation technique. The effect of cell isolation is especially relevant to the primary calvarial cells because these cells are digested out using collagenase. In our unpublished work, we have observed that collagenase treatment changes cell metabolic profile. Cells treated with collagenase for 30 min at 37 °C showed decreased levels of sugars and TCA cycle metabolites in steady state metabolomics detection. From 89 detected metabolites found in non-collagenase treated cells, only 43 were detected when collagenase was used. Additionally, enrichment metabolic pathway analysis revealed that collagenase-treated cells presented a glycolytic shift when compared to non-treated cells. This effect may explain the noted variability in reported metabolic values in primary calvarial cells described above. One possible solution to address this variability is to perform multiparametric studies with a minimum requirement of assessing both glycolysis and OxPhos and using reference cell lines such as for example, 143B (glycolytic) vs hFOB (oxidative).

Another important point to consider is that osteogenic differentiation is characterized by an initial stage of high proliferative rate, and a later postmitotic bone matrix synthesis and deposition stage. As we discussed in the previous sections, proliferation and matrix deposition in OBs require distinct metabolic signaling and energetic profiles. Therefore, different results can be found depending on which stage of osteogenic differentiation cells are being analyzed. Additionally, unsynchronized cells undergoing differentiation can skew or mask the results. OBs are short-lived cells, the caspase-dependent apoptotic program initiates with the dismantling of mitochondrial machinery. Therefore, OBs undergoing apoptosis are expected do downregulate OxPhos use which can also skew some metabolic results. It is important that future experiments account for such considerations and unbiased experimental molecular approaches are applied more often to resolve the disparities in the field.

## 7 Bioenergetic changes observed in bone pathologies

### 7.1 Inflammation

Inflammation is a condition found in various pathologies, such as aging and trauma including bone fracture. Vast literature exists on the role of inflammation in pathogenesis of various bone disorders. However, most of this literature does not consider the effects of inflammation on bone cell bioenergetics. Therefore, we would like to discuss what is known about such effects. We will focus on the major inflammatory signaling molecule, NF-κB. NF-κB is a heterodimeric transcription factor that localizes in the cytosol in complex with the inhibitor, IκB, and translocates into the nucleus upon activation and proteasome-dependent degradation of IκB (Greten and Karin, 2004; Salminen et al., 2008). In the nucleus it binds specific DNA sequences located within the promoters of target genes and regulates expression of these genes (Salminen et al., 2008). Importantly for the field of bioenergetics, NF-κB was also found in mitochondria. Bottero et al. (2001) discovered presence of the NF-κB p65 subunit in the mitochondrial fraction, possibly in association with the mitochondrial adenine nucleotide translocator (ANT). They used several lines of evidence to support this discovery however functional significance of the mitochondrially localized NF-κB remained unknown. Since the primary function of NF-κB is to bind DNA and regulate transcription, it is logical to assume that NF-κB may bind mtDNA and regulate transcription of mtDNA-encoded genes. Cogswell et al. (Bottero et al., 2001) also detected NF-κB in mitochondria and showed that stimulation with TNFα leads to downregulation of mtDNA-encoded cytochrome *c* oxidase III and cytochrome *b* genes; while NF-κB inhibition prevents such an effect. Furthermore, Johnson et al. (2011) not only detected NF-κB presence in mitochondria but also showed direct binding of RelA (p65) NF-κB subunit to mtDNA in the D-loop promoter region and changes in cell respiration after manipulating NF-κB. These data suggest that NF-κB not only resides in mitochondria but may also negatively regulate expression of mtDNA-encoded mitochondrial proteins potentially causing disruption of the mitochondrial respiratory chain. In addition to the effects on mtDNA, there may be mtDNA-independent effects of mitochondrial NF-κB due to its interaction with ANT contributing to mitochondrial dysfunction. The question remains of how such a large complex as NF-κB can enter intact mitochondria. Assuming that it can cross the more permeable outer mitochondrial membrane barrier, crossing the inner mitochondrial membrane barrier seems highly unlikely without major permeabilization of the membrane. This brings into light the phenomenon of the mitochondrial permeability transition (MPT) due to opening of the MPT pore (MPTP) gated by cyclophilin D (CypD) (Giorgio et al., 2010; Bernardi et al., 2021; Petronilli et al., 1999). MPTP opening may facilitate the entry of NF-κB into mitochondria. The role of MPTP in bone cells and bone pathology will be discussed in detail in the next section.

### 7.2 Aging

Bone maintenance is achieved by the appropriate balance between bone formation and resorption. During aging, OB decreased activity, BMSC, OB, and OT senescence, and further cellular pool exhaustion shift this tenuous balance towards bone resorption (Farr and Almeida, 2018; Moerman et al., 2004; Connor et al., 2018). Our lab and others have shown that OB differentiation relies on higher levels of OxPhos activation and mitochondrial bioenergetics, which are compromised during aging (Shum et al., 2016b). In general, mitochondrial function and integrity are impaired during aging in several mechanisms such as, increased accumulation in mtDNA damage and ROS generation, decreased levels of respiratory chain proteins, deficient mitophagy, the quality control tool in mitochondria, dysfunctional UPRmt, and lessened ER-mitochondrial contact and therefore, organelle interaction (Barja, 1999; Sun et al., 2016; Rottenberg and Hoek, 2017; Moehle et al., 2019). ROS increase is also accompanied by a decline in cellular ROS-scavenging capacity leading to increased MPTP opening and loss of integrity of the IMM prompting higher oxidative stress, inflammation, and catabolic pathways. Not surprisingly, aging is by far the major risk factor for chronic and degenerative diseases.

There is vast evidence pointing that the deficient mitochondrial machinery observed in aging poses as an important factor in bone loss and fragility, and manipulation of such aging-related mitochondrial parameters are potential therapeutic targets to ameliorate bone-aging phenotype. Since mitochondria are contain TCA cycle and OxPhos machinery, we would only expect that aging mitochondria lead to important changes in the metabolic profile of aged bones. In fact, our group demonstrated that middle-aged 13 month-old mice present important changes in metabolite levels revealed by metabolomic analysis in long bones, namely a glycolytic shift, mitochondrial dysfunction, and oxidative stress (Shum et al., 2016b). At 18 month of age, mouse bone tissue showed an even higher metabolic shift towards glycolysis accompanied by a decrease in TCA-derived metabolite levels (Shum et al., 2016b). This increase in glycolysis in aging is most likely an adaptation to the impaired mitochondrial function. We also detected an increased energy charge, an important regulator of anabolic/catabolic pathway decision (Bassham and Krause, 1969), and higher oxidized glutathione levels, a marker for oxidative stress (Aquilano et al., 2014). These data indicate that the disturbed metabolic pathway in aged bone tissue is poised to promote bone catabolism and oxidative stress, respectively.

### 7.3 Effects of bone therapies on bone metabolism

As extensively discussed above, energy metabolism and OxPhos activation are important for bone anabolism and homeostasis. Therefore we would only expect that therapies meant to improve OB function would also have an effect on OxPhos regulation and interconnected metabolic pathways. Even though there are neither clinical nor pre-clinical studies depicting how specific FDA-approved treatments impact bone metabolism, we can discuss some relevant published results. The known effects of PTH on bone metabolism are described in Section 4.5. Although it is vastly reported that an intermittent regimen of PTH peptide induces bone anabolism and changes some aspects of tissue metabolism (Kurland et al., 2000; Black et al., 2008; Rendina-Ruedy and Rosen, 2022), the exact effect on bone metabolic pathways is not totally clear. We have previously showed that BMP2 or Wnt3a induce OxPhos via Akt signaling (Smith and Eliseev, 2021), therefore we can assume that therapies using anti-sclerostin or recombinant BMPs should produce some effects on bone metabolic profile as well. Hormone replacement therapy is also described as an efficient treatment to prevent bone loss and decrease the risk fracture in postmenopausal women, although it is not considered the first-option treatment due to concerns of side effects (Levin et al., 2018). In osteoclasts (OCL), estrogen suppresses cytokines such as receptor activator of nuclear factor kappa-Β ligand (RANK-L), macrophage colony-stimulating factor (M-CSF), and Interleukin-6, regulating OCL proliferation, maturation and apoptosis. In OBs, estradiol is shown to induce early differentiation and increase cell lifespan (Kousteni et al., 2001). Not surprisingly, mice with Estrogen Receptor-α deletion in osteoprogenitor *Prx1*-expressing cells is characterized by reduced cortical bone thickness (Almeida et al., 2013). Estrogen plays an important role in cell metabolism by inducing higher OxPhos usage (Gaignard et al., 2017; Torres et al., 2018) and protecting mitochondria against ROS and opening of the mitochondrial permeability transition pore (Burstein et al., 2018). It remains to be elucidated how much of the bone anabolic effect of estrogen is due to its positive effect on OB mitochondria. In sum, there is a growing number of studies showing that specifically targeting mitochondrial function and, thus, OB metabolism can produce beneficial effects and that this venue can be further explored as a therapeutic option to stimulate bone anabolism.

## 8 Methods to study bioenergetics on a subcellular, cellular, and tissue levels

We will only briefly outline (see Table 3) the available techniques to study bone cell and tissue bioenergetics since these techniques are described elsewhere, e.g. in excellent reviews by Brandt and Nicholls (Brand and Nicholls, 2011) and others (Scaduto and Grotyohann, 1999; O'Reilly et al., 2003; Zorova et al., 2018; Hawkins and Davies, 2019; McLaughlin et al., 2020).

**Table 3:** Methods to study bioenergetics. TMRE, tetramethylrhodamine esther; TMRM, tetramethylrhodamine, methyl ester; NBDG, 2-(N-(7-nitrobenz-2-oxa-1,3-diazol-4-yl)amino)-2-deoxyglucose; NAO, nonyl acridine orange; IRDye-2DG, infrared dye 2-deoxyglucose; PET, positron emission tomography; FDG, fluorodeoxyglucose.

Level	Method	Advantages	Limitations
Subcellular	Metabolic enzymes activities: -Glycolytic -Krebs cycle -Mitochondrial respiratory chain	-Easier to perform when compared to live cells -Direct measure of activity	Missing cellular and organellar context and interactions
	Isolated mitochondria: -oxygen consumption (Clark electrode, Oroboros) -membrane potential, ΔΨm (TMRE, TMRM)	-Direct measure of organelle function -Ability to study direct effects of drugs and biologicals on mitochondria	Missing cellular and organellar context and interactions
Live cells	Seahorse XF (glycolysis and OxPhos)	-High throughput analysis of overall cell energy metabolism -Intact cell content and interactions -Ability to study direct cell responses	-Suited mostly for monolayer cultures -Cell density affects readings
	Fluorescent probes: -glycolysis (NBDG) -mitochondrial mass (NAO) -mitochondrial function, ΔΨm (TMRE, TMRM)	-Intact cell content and interactions -Ability to study direct cell responses -Ability to perform multiparametric studies	-Difficult to interpret due to non-linear signal dynamics -Requires sophisticated equipment
	Metabolomics and Metabolic tracing	-Intact cell content and interactions -Ability to dissect various metabolic pathways	-Challenging analytical steps -Requires sophisticated equipment
Tissue	Fluorescence (NBDG and IRDye-2DG)	Ability to assess whole tissue metabolism	Limited number of probes is suitable for whole tissue
	Metabolomics		-Challenging analytical steps -Requires sophisticated equipment
Live animals	Fluorescence (IRDye-2DG)/whole animal imaging	Ability to assess intact metabolism in live animal	-Limited number of probes is suitable for live animals -Requires sophisticated equipment
	PET (FDG)/whole animal imaging		
	NADH autofluorescence/multiphoton microscopy	Ability to assess intact metabolism of individual cells in live animal	-Challenging analytical steps -Requires sophisticated equipment

### 8.1 Subcellular level

#### 8.1.1 Enzyme activities

On a subcellular level, one can biochemically assess activities of individual enzymes, both glycolytic and mitochondrial. Relative changes in enzyme activities can inform about the overall efficiency of the pathway that they belong to. Such assays involve tissue homogenization under the conditions that preserve enzyme conformation and function, e.g. ice-cold bath, absence of proteases, etc. Very often such assays are combined with measurements of expression levels of enzymes or their subunits if they are multiunit complexes. This became especially common after the development of high throughput transcriptomic and proteomic methods (Haider and Pal, 2013). One major weakness of the subcellular approach is that the interpretation of the data is not trivial. First, this approach lacks the cellular context and isolated enzymes placed in an artificial buffer may behave very differently than they do in vivo. Second, there is always some damage due to homogenization. Third, protein expression does not always reflect its activity. For example, higher than normal expression of mitochondrial proteins is often a compensatory measure during mitochondrial dysfunction (To et al., 2019), yet it is sometimes interpreted as a marker of increased mitochondrial activity.

#### 8.1.2 Isolated mitochondria

Another subcellular level of investigation is working with isolated organelles, in our case, mitochondria. Isolation of mitochondria requires conditions that preserve their integrity, such as ice-cold bath, absence of calcium, etc. Functional assays of isolated mitochondria include measurement of basal, ADP-induced, and maximal (uncoupled) oxygen consumption and calculation of a respiratory control ratio. This can be done using Clark type electrode (Silva and Oliveira, 2012) and air-tight temperature-controlled chamber or using more recent Oroboros technique. Another functional assay is the measurement of mitochondrial membrane potential, ΔΨm, using lipophilic cationic probes, such as tetraphenylphosphonium (TPP^+^) and TPP-selective electrode (Moreno et al., 2015) or fluorescent tetramethylrodamine ester (TMRE) and fluorescent detection (Scaduto and Grotyohann, 1999). The same drawbacks as for enzymatic assays apply here, i.e. the lack of cellular context and possible damage during isolation.

### 8.2 Live cells

#### 8.2.1 Extracellular fluxes

Analysis of live cells provides more physiologically relevant information when compared to cell extracts or isolated mitochondria. There are various live cell-based assays of bioenergetics. Of them, one particular method has recently gained popularity – analysis of extracellular fluxes using the Seahorse XF technology now owned by Agilent (Gu et al., 2021). It allows for simultaneous high throughput measurements of OxPhos and glycolytic function under the physiologically relevant conditions. It also allows for automated temporally controlled additions of up to four different compounds during the course of the experiment. The readouts are the oxygen consumption rate (OCR), a measure of OxPhos, and extracellular acidification rate (ECAR), a measure of glycolytic lactate production. This is a very informative method and, in most cases, the assay of choice. However, it still requires a lot of optimization in each particular case and careful consideration of media constituents and conditions. In general, the assay media should be as close to physiological conditions as possible unless there are specific reasons to manipulate them. For example, pyruvate is often added to the media as a fuel, however cells in vivo are exposed to very low levels of pyruvate. If the goal is to measure basal activity levels of OxPhos and glycolysis, exogenous pyruvate can skew these measurements. However, when the goal is to study the effect of forced activation of OxPhos for example by replacing media glucose with galactose (Shares et al., 2018), presence of pyruvate can maximize the effect. Another example is glucose concentration. Many different media formulations have supraphysiological levels of glucose, e.g. 25 mM. Such levels of glucose can induce the Crabtree effect (Pfeiffer and Morley, 2014), i.e. inhibition of OxPhos, and, thus, should be avoided unless the goal is to study diabetes-like conditions. If the Seahorse XF is not available, one can still detect changes in the lactate production as a measure of glycolysis using enzymatic assay and oxygen consumption as a measure of OxPhos using the Clark electrode.

#### 8.2.2 Glycolysis- and mitochondria-specific fluorescent probes

In addition to the Seahorse XF, there are many fluorescence-based methods that allow for a high throughput analysis of live cells, such as flow cytometry. Very few glycolysis-specific probes are available. Among them are NBDG and IRDye 2-DG. They are non-metabolized glucose analogs and can, therefore, measure glucose uptake by the cell (Zou et al., 2005; Zhou et al., 2009). The majority of fluorescent probes are designed to measure mitochondrial membrane potential, ΔΨm, as a relative measure of OxPhos or morphology (networked or fragmented). Such probes are membrane-permeable lipophilic cations that accumulate in the cytosol and mitochondria according to the Nernst equation (Ross et al., 2006). The mitochondrial inner membrane is highly charged (−180 mV, negative inside) due to a large gradient of protons. The Nernstian behavior results in accumulation of the probe in mitochondria at 1000 times higher concentration than in the cytosol. Therefore, these probes produce a very bright mitochondrial signal with the wide choice of colors/wavelengths. However, careful consideration should be given to choosing the right probe. We again refer the reader to the previous reviews for the specifics (Borle et al., 1960; Scaduto and Grotyohann, 1999) while summarizing the probes of choice. For the dynamic ΔΨm measurements, e.g. ΔΨm changes after additions of drugs or biologics, TMRE and TMRM are preferred probes. At low concentrations of less than 20 nM, their fluorescence is in direct dependence of the ΔΨm value, i.e. the higher the ΔΨm, the higher the signal. At higher concentrations, they are in the so-called ‘quench’ mode or inverse dependence of the ΔΨm value, i.e. the lower the ΔΨm, the higher the signal (Scaduto and Grotyohann, 1999). An uncoupler, such as FCCP, should be used with these probes as a negative control, sometimes together with the ATPase inhibitor, oligomycin, because uncoupled mitochondria may use their Complex V ATPase and ATP to rebuild the ΔΨm (see Table 3).

If non-dynamic measurements are done, e.g. comparing ΔΨm values between different groups of cells at baseline, CMXRos and DiOC6(3) probes offer sufficient reliability (Borle et al., 1960; Le et al., 2006). Once taken up by the cells, these probes are also relatively resistant to cell manipulations, such as freezing, fixation, and uncoupling. Thus, an uncoupler for negative control should be loaded before CMXRos and DiOC6(3). We have successfully used CMXRos to measure oxidative metabolism in fresh BMSCs from human bone marrow aspirates (Shum et al., 2020). One other important consideration here is that the mitochondrial mass may differ between different samples and normalization should be done when measuring whole cell and not individual mitochondria fluorescence. There are various MitoTrackers that specifically label mitochondria, such the MitoTracker Red or Green, as well as mitochondria-specific membrane-labeling probes, such as nonyl acridine orange (NAO). All these mitochondrial mass probes do, however, show some dependence on the ΔΨm (Borle et al., 1960) and, thus, not ideal. Another possibility is to normalize to mtDNA (Memon et al., 2017) or mitochondrial protein (VDAC, cytochrome oxidase, etc) levels. The best strategy is to use several methods of normalization.

#### 8.2.3 Metabolomics

It is not our intent to discuss the advantages and weaknesses of each metabolomic method in regards of small metabolite separation and detection but to present some overall applications to study metabolism in osteogenic cells and bone tissue. In general, metabolomic data allow a very broad analysis of carbon metabolism and its interconnected pathways and more importantly, it provides a quantitative approach for characterization of metabolic profile and phenotype in cells (Cakmak et al., 2012). As an example, we work with parameters to detect 150 cellular metabolites including 6- and 3-carbon glycolytic, PPP, and TCA cycle intermediates as well as nucleotides, redox compounds, and key amino acids (Shum et al., 2016b). Further calculations can be obtained to characterize the oxidative state of the cell, pyridine balance, and energy charge status. Of note, the typical metabolomics run cannot resolve all cellular metabolites at once, but still able to detect a broad representation. More specific pathways can be targeted with higher resolution, but it requires further optimization in detecting the metabolites of interest among the total pool. Additionally, most of metabolic steps are reversible and reactions can go in the opposite direction. Therefore, the steady-state metabolic panel can provide only a quantitative representation of the metabolite pool “frozen in time”; and it cannot be interpreted as a directional flowchart.

^13^C metabolic flux analysis, however, utilizes stable isotope tracing and allows a metabolic network analysis providing directionality (Antoniewicz, 2018). ^13^C-Glucose and ^13^C-Glutamine are the most utilized tracers for metabolic analysis and can be used individually or in parallel labeling experiments. Labeled substrates are metabolized; and carbon rearrangement in downstream reactions provides specific labeling patterns which can be measured to determine the metabolic flux. This approach is fundamental in understanding how metabolic flux affects cell function and should be more broadly applied in the study of bone cells. Other stable isotopes such as ^2^H, ^18^O, and ^15^N can also be used for metabolic flux analyses. However, understanding the advantages and drawbacks of each tracer method is crucial for its application and correct interpretation.

There are several technical details that are important to remember. Gas (GC) or high-performance liquid chromatography (HPLC) is the most usual methods used for metabolite separation, which requires special attention during sample collection. Both processes utilize methanol for metabolite extraction and therefore, a destructive method that requires the use of intact cells, without prior lysis, and incubation in very cold temperatures (−80 freezer, dry ice, or liquid nitrogen). Following extracts drying and resuspension, samples are run into the separation step (GC or HPLC) and detected by mass spectrometry. Another common detection method is the nuclear magnetic resonance (NMR) spectroscopy. NMR is the only detection method that does not require prior metabolite separation, allowing sample recovery for extra experiments (Larive et al., 2015).

Once the data are generated, several analyses can be performed and there are plenty of web-based free software available (Xia and Wishart, 2016; Cambiaghi et al., 2017; Chong and Xia, 2020; Pang et al., 2021). But additional calculations are needed to evaluate the bioenergetic and redox states of the cell. NAD^+^/NADH ratio and NADPH level are important indicators of redox balance (Stein and Imai, 2012; Xiao et al., 2018). Decreased values compared to control are suggestive of energetic stress and diminished functional capacity of ROS scavenging, respectively. Another important indicator of cellular oxidation status is the reduced to oxidized glutathione (GSH/GSSG) ratio (Pizzorno, 2014). Glutathione can prevent damage caused by ROS detoxifying hydrogen peroxide generated by superoxide dismutase 2. It is expected that in healthy cells and tissue, the majority of total glutathione pool is in the reduced form. Therefore, decreased GSH/GSSG ratio is an important indicator of oxidative stress. High and sustained levels of oxidative stress can lead to mitochondrial dysfunction and trigger cell catabolic pathways. Considering the 1st law of thermodynamics and the impact of Gibbs free energy and absolute equilibrium constant in the direction of an enzymatic reaction, the levels of high energy nucleotides can be used to infer about the overall metabolic status of the cell. Energy charge that is calculated by the formula (ATP + ADP/2) / ATP + ADP + AMP, can provide an important picture of the cell metabolic status. Elevated energy charge is an indicator of compromised mitochondrial function and progressive decline in oxidative metabolism (Shum et al., 2016b). Additionally, high energy charge is known to inhibit catabolic (ATP-generating) pathways and stimulate anabolic (ATP-utilizing) pathways. Although time-consuming, metabolomic analyses can generate an enormous amount of data and provide a deep investigation into the cell metabolic decisions and pathways.

### 8.3 Live and freshly isolated tissue

Measuring bioenergetic parameters in vivo remains very challenging. The available methods rely on sophisticated techniques that are not always readily available, such as the multiphoton microscopy.

#### 8.3.1 Imaging techniques

^18^F-fluorodeoxyglucose (FDG) positron emission tomography (PET) often combined with computed tomography (CT) allows detection of glucose metabolism in live animal tissues including bone. FDG PET/CT has been successfully used before to measure glucose uptake by different bones (Mittra and Iagaru, 2010; Costelloe et al., 2014). The downside of it is the high cost of the equipment (PET scanner). There are alternatives to PET that provide the same information, such as using the IVIS imager to detect signal from fluorescence-labeled deoxyglucose, NBDG or IRDye 2-DG (Zhou et al., 2009) described above. If the imager is not available, freshly prepared frozen tissue sections from NBDG- or IRDye 2-DG-injected animals can be analyzed using fluorescence microscopy (Xie et al., 2012). Freshly isolated cells from the same animals can be analyzed with flow cytometry (Palmer et al., 2016). In principle, the same approach can be used to detect mitochondrial function using potentiometric probes that can sustain cell or tissue isolation procedure, such as CMXRos and DiOC6(3) described above. The limitation here is that it is challenging to have a negative control sample usually required for these mitochondrial probes. One would need to inject animals not only with the probe but also with the uncoupler or mitochondrial inhibitor that will undoubtedly be toxic to the animal.

With recent improvements in microscopy, it has become easier to image cells and tissues in live animals. Multiphoton microscopes allow for imaging of not only cells on the surface but also cells residing deeper within the tissue (Ustione and Piston, 2011). This technique can detect not only fluorescently labeled cells but also autofluorescence from such compounds as NADH. NADH autofluorescence has been used to measure cell bioenergetics, mostly as a measure of oxidative pathway (Schaefer et al., 2019). However, there is now convincing data showing that different forms of NADH, free vs protein-bound, reflect cytosolic (mostly glycolysis-generated) vs mitochondrial (OxPhos-generated) NADH, respectively (Schilling et al., 2022). These different forms of NADH have different fluorescence decay times (τ), which can be detected via fluorescence lifetime imaging (FLIM). This technique has been recently employed to assess bioenergetics of OBs in different regions of the bone and at different stages of differentiation (Borle et al., 1960).

#### 8.3.2 Metabolomics

To our knowledge, we are the only group that have published metabolomic data from in vivo bone samples (Shum et al., 2016b). Other than utilizing bone samples for metabolite extraction, in vivo metabolomic run is similar to the one using in vitro samples. Tissue preparation requires bone tissue to be rapidly processed and cleaned of soft tissue, cartilage, and periosteum. Bone marrow is flushed out and bone shafts are flash frozen in liquid nitrogen. Equal quantities of tissue are pulverized in liquid nitrogen and metabolites extracted in methanol. The correct interpretation of results requires supplementary experiments to account for potential changes in distinct cell populations within the bone. Although the heterogeneous nature of cells analyzed by this approach can limit its use, well designed and controlled manipulations can successfully provide good quality data enhancing our understanding of cell metabolism in bone tissue.

^13^C metabolic flux analysis in vivo is a tool vastly used in cancer studies in both animal and human subjects. Using the same approach of stable-isotope tracing in culture, in vivo flux analysis differs by the fact of requiring intravenous infusion of ^13^C and later tissue collection. There are no established protocols for ^13^C infusion regimen targeting bone tissue but, intra-operative ^13^C-glucose infusions in humans subjects showed sufficient tracing resolution in osteosarcoma samples (Johnston et al., 2021). This suggests that in vivo flux analysis can potentially be used to perform metabolic tracing in bone tissue, having an important applicability in understanding how different mouse models and signaling manipulations can impact bone metabolism (Sanchez-Arago et al., 2013; Lee et al., 2019; Hollenberg et al., 2020; Pattappa et al., 2011; Forni et al., 2016).

## 9 Conclusions

• Osteogenic lineage cells are plastic, utilizing different fuels and energetic pathways depending on their differentiation status, environment, and tissue needs
• Like other stem cells, osteoprogenitors are glycolytic. OxPhos is activated in the process of osteogenic differentiation
• While early works defined osteoblasts as mostly glycolytic, the majority of recent works indicate that they are heavily dependent on OxPhos
• There is a crosstalk and mutual regulation between the metabolic machinery and various signaling and gene regulatory mechanisms in osteolineage cells
• Bone aging is associated with the glycolytic shift, mitochondrial dysfunction, and oxidative stress
• Multiparametric functional analysis is required to assess cell and tissue bioenergetics

## Declaration of competing interest

The authors declare no competing interests.
